# Efficacy of traditional Chinese exercise for obesity: A systematic review and meta-analysis

**DOI:** 10.3389/fendo.2023.1028708

**Published:** 2023-03-01

**Authors:** Ze Yang, Kai Huang, Yang Yang, Qike Xu, Qiaofeng Guo, Xiang Wang

**Affiliations:** ^1^ The First Clinical Medical College, Zhejiang Chinese Medical University, Hangzhou, China; ^2^ Department of Orthopedics, Tongde Hospital of Zhejiang Province, Hangzhou, China; ^3^ Department of Traditional Chinese Medicine, Neighborhood Good Doctor No. 6 Street Clinic, Hangzhou, China

**Keywords:** obesity, traditional Chinese exercise, Tai Chi, Qigong, Baduanjin, Wuqinxi, COVID-19 lockdown, systematic review and meta-analysis

## Abstract

**Background:**

Obesity is considered one of the biggest public health problems, especially in the background of the coronavirus disease 2019 (COVID-19) lockdown. It is urgent to find interventions to control and improve it. We performed this systematic review and meta-analysis to summarize the effect of traditional Chinese exercise on obesity.

**Methods:**

We searched PubMed, Embase, Cochrane Library, the China National Knowledge Infrastructure (CNKI), the Chinese Scientific Journal Database (VIP), the Chinese Biomedical Literature Database (CBM), and WanFang database for updated articles published from the inception of each database to June 2022. Randomized controlled trials (RCTs) on traditional Chinese exercise in weight reduction were included, and related data were extracted. The random-effects model was used to adjust for the heterogeneity of the included studies, and funnel plots were used to examine publication bias.

**Results:**

A total of 701 participants were included in the 10 studies. Compared with the control group, the outcome of body weight [mean difference (MD) = −6.10; 95% CI = -8.79, -3.42], body mass index (MD = −2.03; 95% CI = -2.66, -1.41), body fat mass (MD = −3.12; 95% CI = -4.49, -1.75), waist circumference (MD = −3.46; 95% CI = -4.67, -2.24), hip circumference (MD = −2.94; 95% CI = -4.75, -1.30), and waist-to-hip ratio (MD = −0.04; 95% CI = -0.06, -0.03) in the intervention group had significant differences. Egger’s test and funnel plots showed that the potential publication bias of the included studies was slight (p = 0.249).

**Conclusion:**

Traditional Chinese exercise is an effective treatment for obesity; people under the COVID-19 lockdown could do these exercises to control weight. However, a precise and comprehensive conclusion calls for RCTs on a larger scale with more rigorous designs considering the inferior methodological quality and limited retrieved articles.

**Systematic review registration:**

www.crd.york.ac.uk/PROSPERO/, identifier CRD42021270015.

## 1 Introduction

In the past 50 years or so, the prevalence of obesity has increased worldwide, reaching an epidemic level (1). The prevalence of obesity has doubled worldwide since 1980 (2). Nearly a third of the global population has been determined to be obese or overweight (3). Since December 2019, when the coronavirus infection first emerged (4), this challenge faced by individuals has been more outstanding, with more than 50% of individuals with obesity reporting increased weight during the lockdown (5). The health problem of obesity ranges from psychological consequences to physical consequences, such as obstructive sleep apnea and arthritis, which severely affect the quality of life; the condition becomes even worse if other diseases occur together with it (6, 7). These diseases include hypertension (8), dyslipidemia, cardiovascular disease, type 2 diabetes mellitus, and dementia (9, 10). Therefore, weight reduction and weight loss maintenance are particularly important.

Studies have manifested that physical exercise is an effective way to achieve weight loss whether in women or in children (11–14). However, many kinds of physical exercise are intense or monotonous, making it difficult for people to maintain exercising. Moreover, patients with obesity cannot do too much intensive and strenuous exercise or it may induce severe exercise-induced muscle injury (15), thus the exercise intensity and duration for obese patients must be carefully selected (16). Traditional Chinese exercise (TCE), originating from traditional Chinese medicine tracing back to approximately 3,000 years ago, is used as a therapeutic and aerobic exercise, including tai chi, qigong, Baduanjin, Wuqinxi, Yijinjing, and other mind–body therapies (17, 18). This kind of exercise is part of low- to moderate-intensity aerobic exercises, which are different substantially from the high-intensity physical exercise (19).

Although meta-analyses have shown the effectiveness of TCE in knee osteoarthritis, stroke, and cognitive outcomes for older adults (20–22), whether TCE is more effective in weight reduction and weight loss maintenance than other exercise interventions or without treatment has not been reported yet. The main objective of this study was to identify whether TCE is effective in weight reduction and weight loss maintenance, to incorporate all available randomized controlled trials (RCTs), and to investigate relevant subgroups.

## 2 Materials and methods

### 2.1 Data source

We searched PubMed, Embase, Cochrane Library, the China National Knowledge Infrastructure (CNKI), the Chinese Scientific Journal Database (VIP), the Chinese Biomedical Literature Database (CBM), and WanFang Database for updated articles published from the inception of each database to 1 June 2022. At the same time, we searched for gray literature that met the inclusion criteria in Open Grey, Clinical Trials.gov, and WHO Clinical Trial Registration Center. When duplicate publications were identified, we chose the most complete and recent trial. Two investigators (YY and ZY) independently retrieved all related studies in the database and excluded duplicate publications. The search strategy for PubMed is presented in **Table 1**.

**Table 1:** Search strategy in PubMed database.

Number	Search terms
#1	Obesity [MeSH Terms]
#2	Obese [Title/Abstract]
#3	#1 OR #2
#4	Traditional Chinese exercise [Title/Abstract]
#5	Tai chi [Title/Abstract]
#6	Tai ji [Title/Abstract]
#7	Qigong [Title/Abstract]
#8	Baduanjin [Title/Abstract]
#9	Liuzijue [Title/Abstract]
#10	Yijinjing [Title/Abstract]
#11	Wuqinxi [Title/Abstract]
#12	#4 OR #5-11
#13	randomized controlled trial [All field]
#14	randomly [All field]
#15	controlled clinical trial [All field]
#16	randomized [All field]
#17	random allocation [All field]
#18	placebo [all field]
#19	single-blind method [All field]
#20	double-blind method [All field]
#21	trials [All field]
#22	comparators
#23	allocation
#24	#13 OR #14-23
#25	#3 AND #12 AND #24

### 2.2 Inclusion and exclusion criteria

Our meta-analysis is reported in line with the Preferred Reporting Items for Systematic Reviews and Meta-Analyses (PRISMA) (23) Statement and has been registered at the International Prospective Register of Systematic Reviews (number: CRD42021270015).

The inclusion criteria are as follows: 1) the studies must be RCTs; 2) age of participation >18 years; 3) diagnostic criteria for obesity in the study are employed according to the region and ethnic group (Asia-Pacific ≥25.0 kg/m^2^ and America ≥30.0 kg/m^2^ for adults); 4) the intervention group must do TCE, such as tai chi, qigong, Baduanjin, Yijinjing, or Wuqinxi, whether combined with other interventions like control group or not. The control group does casual exercise or only diet education, auricular plaster therapy, acupoint catgut-embedding therapy without exercise, etc.

Exclusion criteria are as follows: 1) the participation according to the region and ethnic group did not meet the body mass index (BMI) of obesity; 2) age of participation ≤18 years; 3) the intervention group did not do TCE or the control group also did TCE.

### 2.3 Study selection and data extraction

Two independent researchers (YY and ZY) read the title, abstract, and full text, screened the literature according to the inclusion and exclusion criteria, and cross-checked the results. If there is a disagreement, a third researcher (QX) will be consulted. Data extracted from the included literature included the following: first author, publication time, mean age or age range, sample size of the study, measures, intervention group and control group taken, duration time, and outcomes.

### 2.4 Quality assessment

The quality of the included studies was assessed by the revised Cochrane risk of bias tool (ROB 2.0) in the Cochrane Handbook for Systematic Reviews of Interventions. The following parameters were evaluated: randomization process, deviations from intended interventions, missing outcome data, measurement of the outcome, and selection of the reported result. Each item was determined to be at high risk of bias, some concerns (unclear risk of bias), or low risk of bias. The combined evaluation of the above items resulted in overall bias.

### 2.5 Statistical analysis

Review Manager software (Revman 5.4 Cochrane Collaboration) was used to meta-analyze the selected studies. The odds ratio (OR) was used as the effect size index for dichotomous variables, and the mean difference (MD) was used as the effect size index for continuous variables, with 95% confidence intervals (CIs) in forest plots. Sensitivity analysis and subgroup analysis will be used to analyze the source of heterogeneity. For the sensitivity analysis, it can help find the source of heterogeneity by reestimating the combined effect using the one-by-one elimination method. For subgroup analysis, the studies may be divided into different subgroups according to the duration time, different types of TCE, etc., and whether it could be shown that the subgroup factors were the source of heterogeneity. When high heterogeneity exists (p < 0.1 or I^2^ > 50%), a random-effects model was used for meta-analysis. Publication bias of major outcome indicators was analyzed by funnel plot in RevMan 5.4 software. When the funnel plot was symmetrical on both sides, the possibility of publication bias between studies was considered low.

### 2.6 Grading of evidence quality

The quality of evidence was graded according to the GRADE method and was classified as high grade, moderate grade, low grade, and very low grade. The assessment criteria used in the GRADE method included risk of bias, inconsistency, indirectness, imprecision, and publication bias. A grade would be given for each outcome.

## 3 Results

### 3.1 Search results

**Figure 1:**
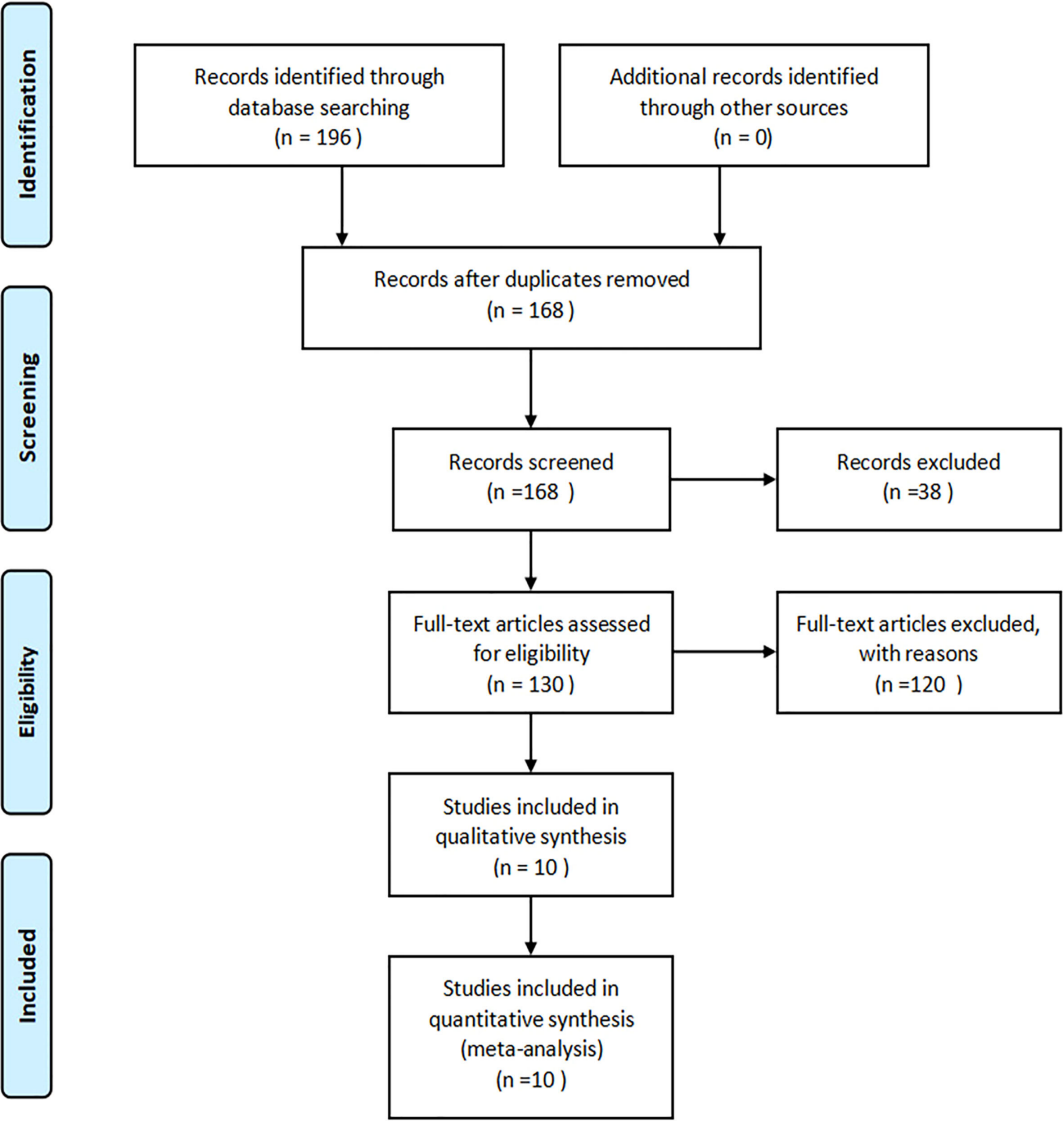
Flowchart of the study selection process.

Our initial search yielded 196 articles in total, 28 of which were removed because of duplication. After screening the titles and abstracts, a further 38 items were taken away. A total of 130 articles were reviewed, among which 10 were included in this meta-analysis (24–33). No further study was identified by manual search. The flow diagram of study selection was shown in **Figure 1** (guideline flow diagram).

### 3.2 Study characteristics

Ten studies with 701 participants were included for the meta-analysis. Outcomes of the studies are as follows: body weight (BW), BMI, body fat mass (BFM), waist circumference (WC), hip circumference (HC), and waist-to-hip ratio (WHR). The main characteristics of the 10 articles were summarized in **Table 2**.

**Table 2:** Characteristics of the 10 studies included in the meta-analysis.

Study	Mean age or age range (AVG)	Sample size (IG/CG)	Intervention Group	Control Group	Duration time	Outcomes
Beebe2013 (25)	IG:60.4 ± 6.2 CG:62.6 ± 5.9	26(13/13)	Tai chi 45 min/week and diet education 45 min/week	diet education 45 min/week	16w	①②③④⑤
Song2015 (26)	IG:59.6 ± 4.72 CG:60.3 ± 4.50	30(15/15)	Tai chi 2×40 min/day and auricular plaster therapy 3-5×5-10 min/day	Auricular plaster therapy 3-5times/day	180d	②③
Fang2018 (27)	18-20	80(40/40)	Wuqinxi 3×45 min/week	Casual exercise 3×45 min/week	one semester	②③④⑤⑥
Li2015 (28)	60-70	60(30/30)	Wuqinxi 5-6×30 min/week	None	6m	①②④
Pan2013 (29)	IG:37.21 ± 8.81 CG:35.28 ± 8.87	64(32/32)	Baduanjin 14×30 min/week and acupoint catgut-embedding therapy twice/month	Acupoint catgut-embedding therapy twice/month	12w	①②④⑤⑥
Wang2015 (30)	19-26	51(23/28)	Yijinjing 3×60 min/week	None	10w	①②③
Yu2013 (31)	40-70	104(52/52)	Baduanjin 3-4times/week and Knowledge education twice a month	Knowledge education twice a month	12m	②⑥
Yu2017 (32)	18-23	46(23/23)	Baduanjin and qigong 5×60min/week	None	16w	①②④⑤⑥
Zhang2011 (33)	IGM:21.34 ± 1.21 IGW:21.55 ± 1.49 CGM:21.17 ± 1.52 CGW:20.86 ± 1.34	40(20/20)	Tai chi 5×30 min/week	None	20w	①②③
Zou2013 (24)	IG:57.42 ± 6.67 CG:57.52 ± 6.20	200(100/100)	Yijinjing 14×30 min/week and health education	Health education	6m	①②④

IG, intervention group; CG, control group; IGM, intervention group man; IGW, intervention group woman; CGM, control group man; CGW, control group woman; w, week; m, month; d, day; min, minute. ①BW, body weight; ②BMI, body mass index; ③BFM, body fat mass; ④WC, waist circumference; ⑤HC, hip circumference; ⑥WHR, waist-to-hip ratio.

### 3.3 Quality of the evidence

**Figure 2:**
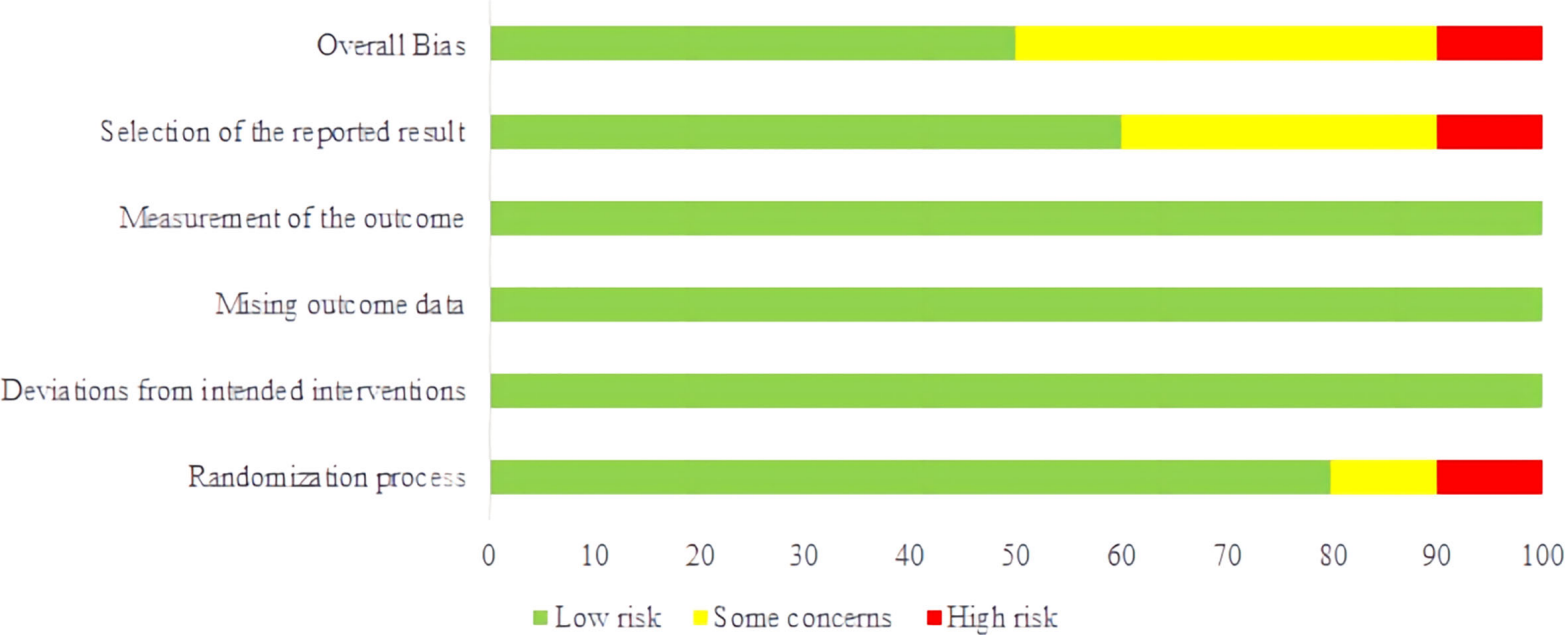
Risk of bias graph. (TCE) on body weight (BW).

Results of risk of bias assessment for all included studies were summarized in **Figure 2**. For bias from the randomization process, eight RCTs were considered as low risk, one study as unclear risk, and one study as high risk. For bias from deviations from intended interventions, bias from missing outcome data, and bias from measurement of the outcome, all 10 RCTs were considered as low risk. For bias from selection of the reported result, six RCTs were rated as likely low risk, three studies were considered as unclear risk, and one study as high risk. For overall risk of bias, five RCTs were considered as low risk, four studies as unclear risk, and one study as high risk. The items that generated bias included the following: ① randomization was reported or but the process was not specifically described or the randomization method was incorrect; ② whether allocation concealment was implemented for the randomization protocol was not reported or unclear; ③ whether blinding was implemented was not clearly reported; ④ whether study protocol registration was not reported or the discussion of reported entries was unclear.

### 3.4 Outcome measures

#### 3.4.1 Body weight

Seven studies (24, 25, 28–30, 32, 33) involving 487 participants reported the outcome of BW. The synthesized data indicated that the TCE group had effectively decreased weight as compared with that of the control group (MD = −6.10; 95% CI = -8.79, -3.42), with a high heterogeneity (I^2^ = 70%, p = 0.0008).

**Figure 3:**
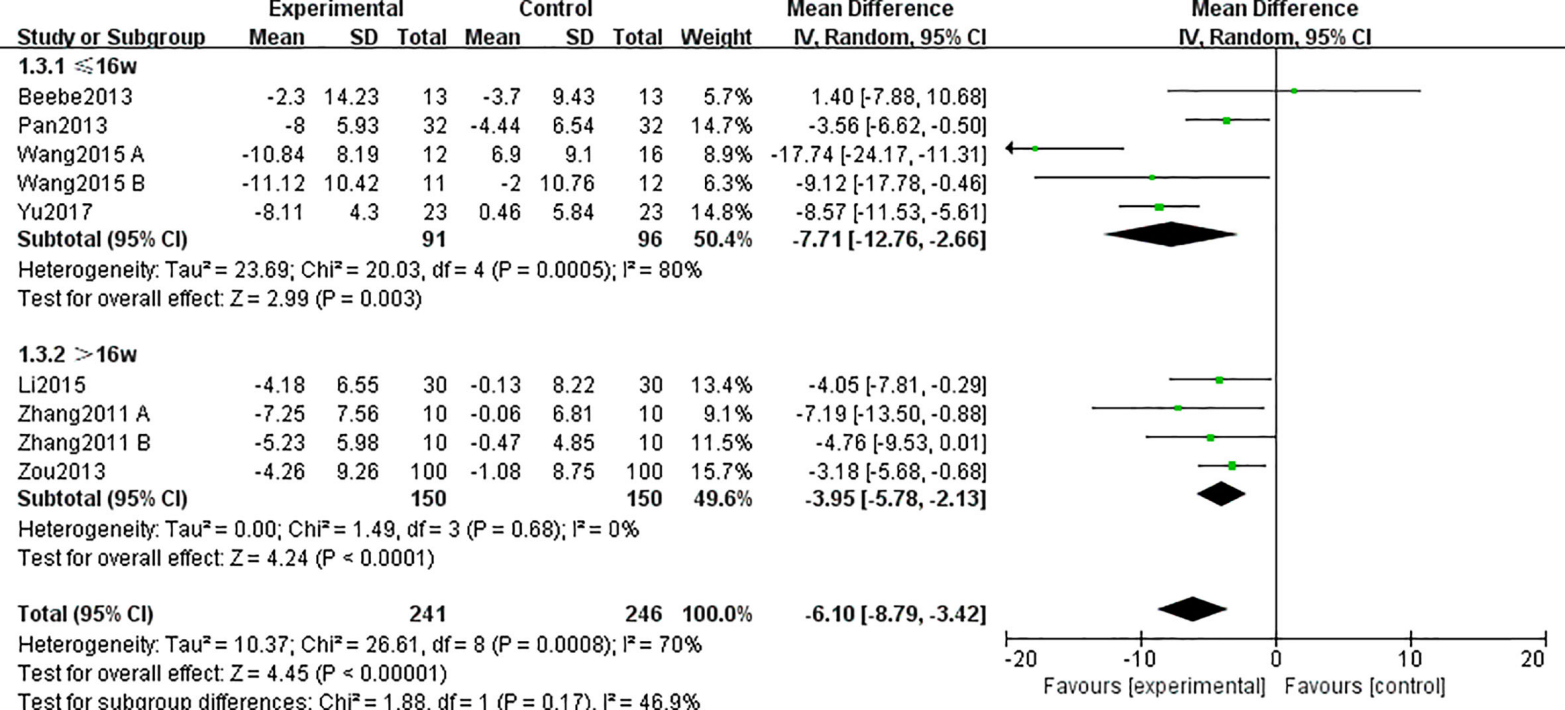
Forest plots and subgroup analysis of duration time for TCE on BW.

Subgroup analysis showed that the heterogeneity in BW between the two groups disappeared when we excluded the studies whose duration time was less than 16 weeks [including three studies (24, 28, 33); MD = -3.95; 95% CI = -5.78, -2.13; I^2^ = 0%] (**Figure 3**). However, whether the duration time was less than 16 weeks or more, there was a significant difference between the two groups in BW.

**Figure 4:**
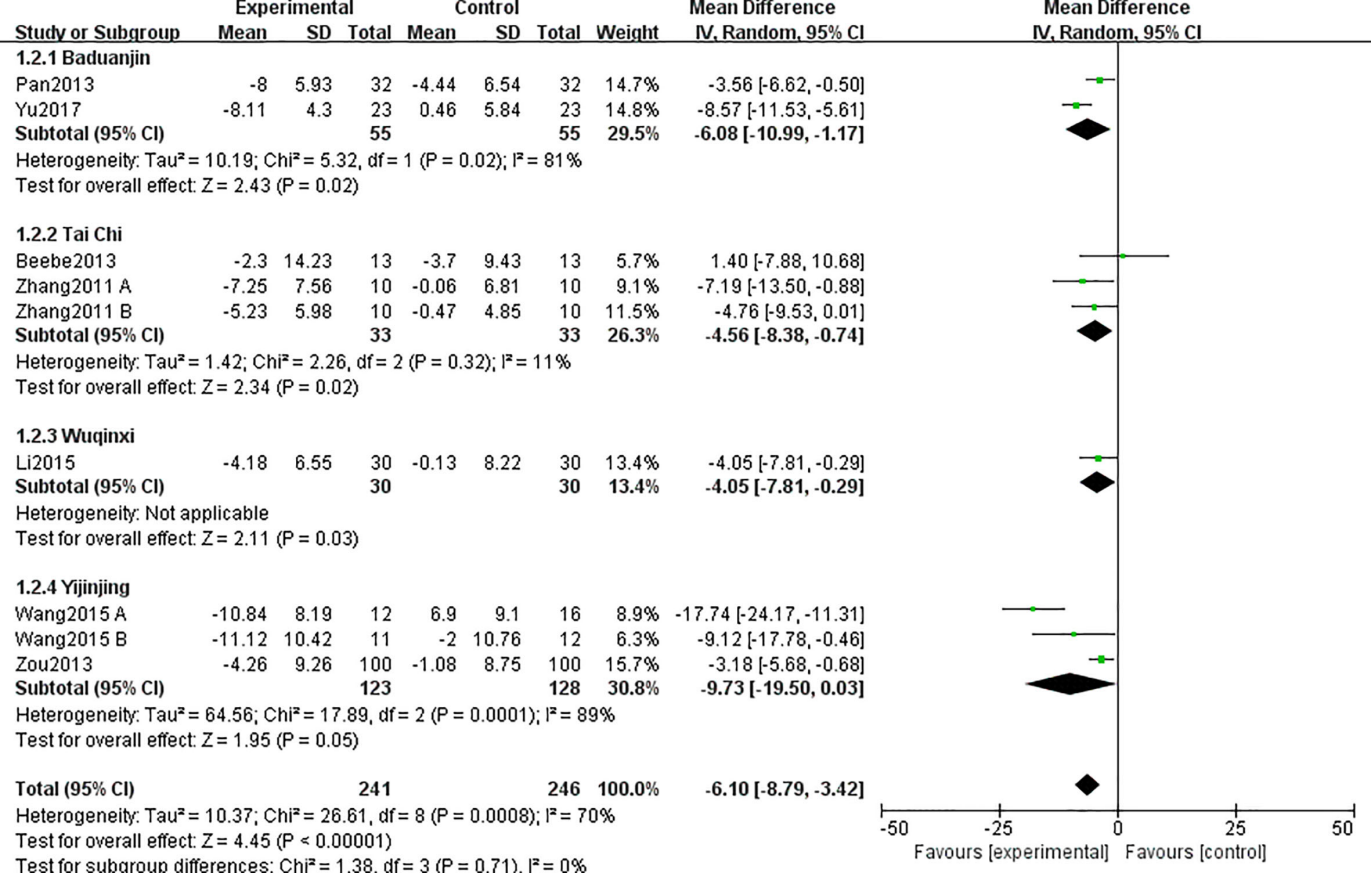
Forest plots and subgroup analysis of different exercise types for TCE on BW.

Subgroup analysis also showed that all kinds of TCE have a significant difference in BW (**Figure 4**), which was consistent with the results reported in every study. Heterogeneity analysis suggested that there was a high heterogeneity in the study of exercise styles of Baduanjin (I^2^ = 81%, p = 0.02) and Yijinjing (I^2^ = 89%, p = 0.0001) (**Figure 4**). It may be due to the existence of low-quality research or differences in the duration and time of the research.

#### 3.4.2 Body mass index

All 10 included studies (24–33) totaling 701 participants were included in this outcome. The synthesized data indicated that the TCE group had a significant impact on decreasing BMI as compared with the control group (MD = −2.03; 95% CI = -2.66, -1.41), and there was a substantial heterogeneity for this synthesized outcome (I^2^ = 64%, p = 0.0001). Therefore, these studies were combined using the random-effects model.

**Figure 5:**
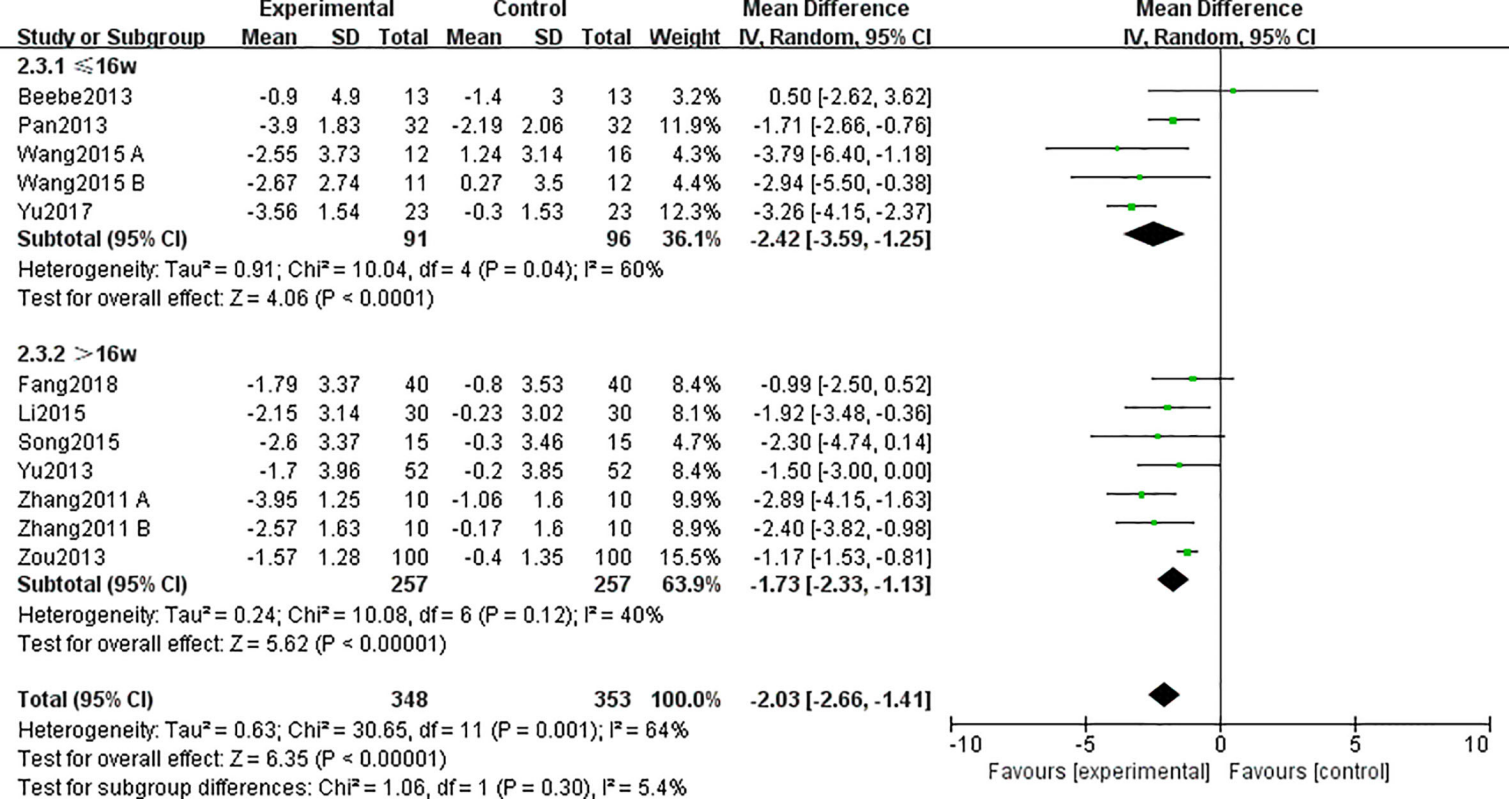
Forest plots and subgroup analysis for traditional Chinese exercise (TCE) on body mass index (BMI).

Subgroup analysis showed that when excluding the studies whose duration time was less than 16 weeks, the heterogeneity in BMI between intervention groups and control groups decreased [including six studies (24, 26–28, 31, 33); MD = -1.73; 95% CI = -2.33, -1.13; I^2^ = 40%; p = 0.12] (**Figure 5**).

#### 3.4.3 Body fat mass

**Figure 6:**
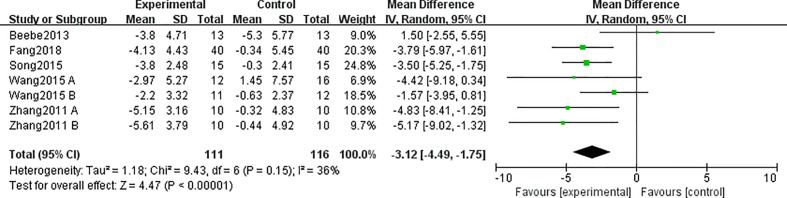
Forest plots for traditional Chinese exercise (TCE) on body fat mass (BFM).

A total of five studies (25–27, 30, 33) involving 227 participants reported the effects of different interventions on the outcome of BFM. The TCE group had effectively decreased it as compared with that in the control group (MD = −3.12; 95% CI = -4.49, -1.75; I^2^ = 36%; p < 0.00001) (**Figure 6**), and there was a low heterogeneity.

#### 3.4.4 Waist circumference

**Figure 7:**
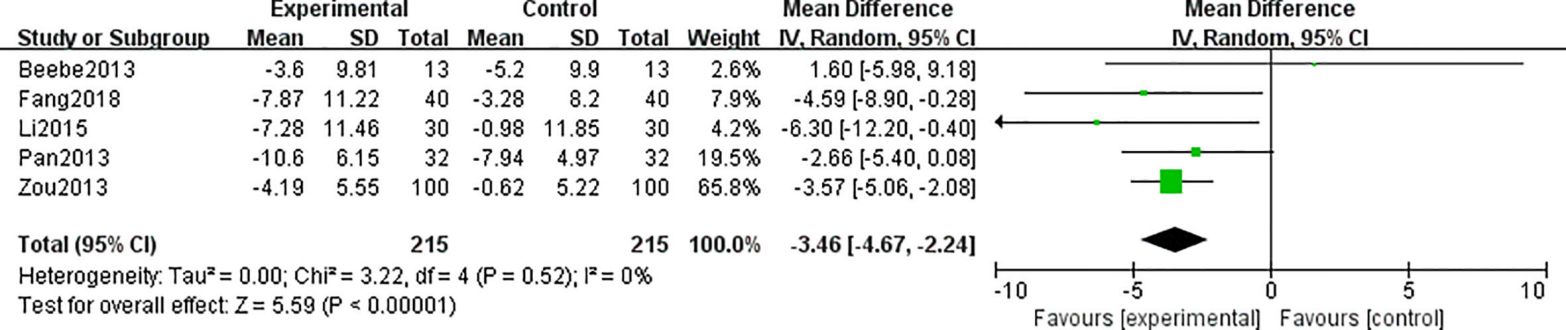
Forest plots for traditional Chinese exercise (TCE) on waist circumference (WC).

A total of six studies (24, 25, 27–29, 32) reported this outcome. Heterogeneity analysis suggested that there was a high heterogeneity (I^2^ = 86%, p = 0.003). The sensitivity analysis was carried out one by one, and it was found that when the research by Yu (32) was removed, the heterogeneity between the studies disappeared (I^2^ = 0%, p = 0.52). The data analysis indicated that WC in the TCE group was lower than that in the control group (MD = −3.46; 95% CI = -4.67, -2.24) (**Figure 7**).

#### 3.4.5 Hip circumference

**Figure 8:**
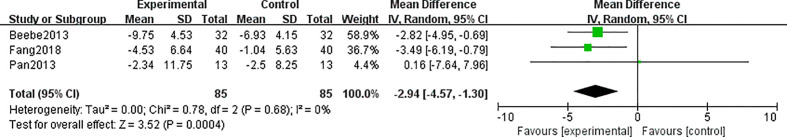
Forest plots for traditional Chinese exercise (TCE) on hip circumference (HC).

A total of four studies (25, 27, 29, 32) involving 216 participants reported the effects of different interventions on the outcome of HC. There was a high heterogeneity (I^2^ = 70%, p = 0.002). Sensitivity analysis suggested that after removing the research by Yu (32), the heterogeneity disappeared (I^2^ = 0%, p = 0.68). The data indicated that the TCE group had an effect in decreasing HC as compared with that in the control group (MD = −2.94; 95% CI = -4.75, -1.30) (**Figure 8**).

#### 3.4.6 Waist-to-hip ratio

**Figure 9:**
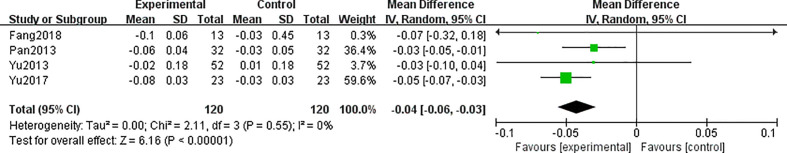
Forest plots for traditional Chinese exercise (TCE) on waist-to-hip ratio (WHR).

Four studies (27, 29, 31, 32) involving 240 participants were included in this outcome. Compared with that in the control group, the TCE group had effectively decreased WHR (MD = −0.04; 95% CI = -0.06, -0.03), with no heterogeneity among the studies (I^2^ = 0%, p < 0.00001) (**Figure 9**).

### 3.5 Evaluation of publication bias

**Figure 10:**
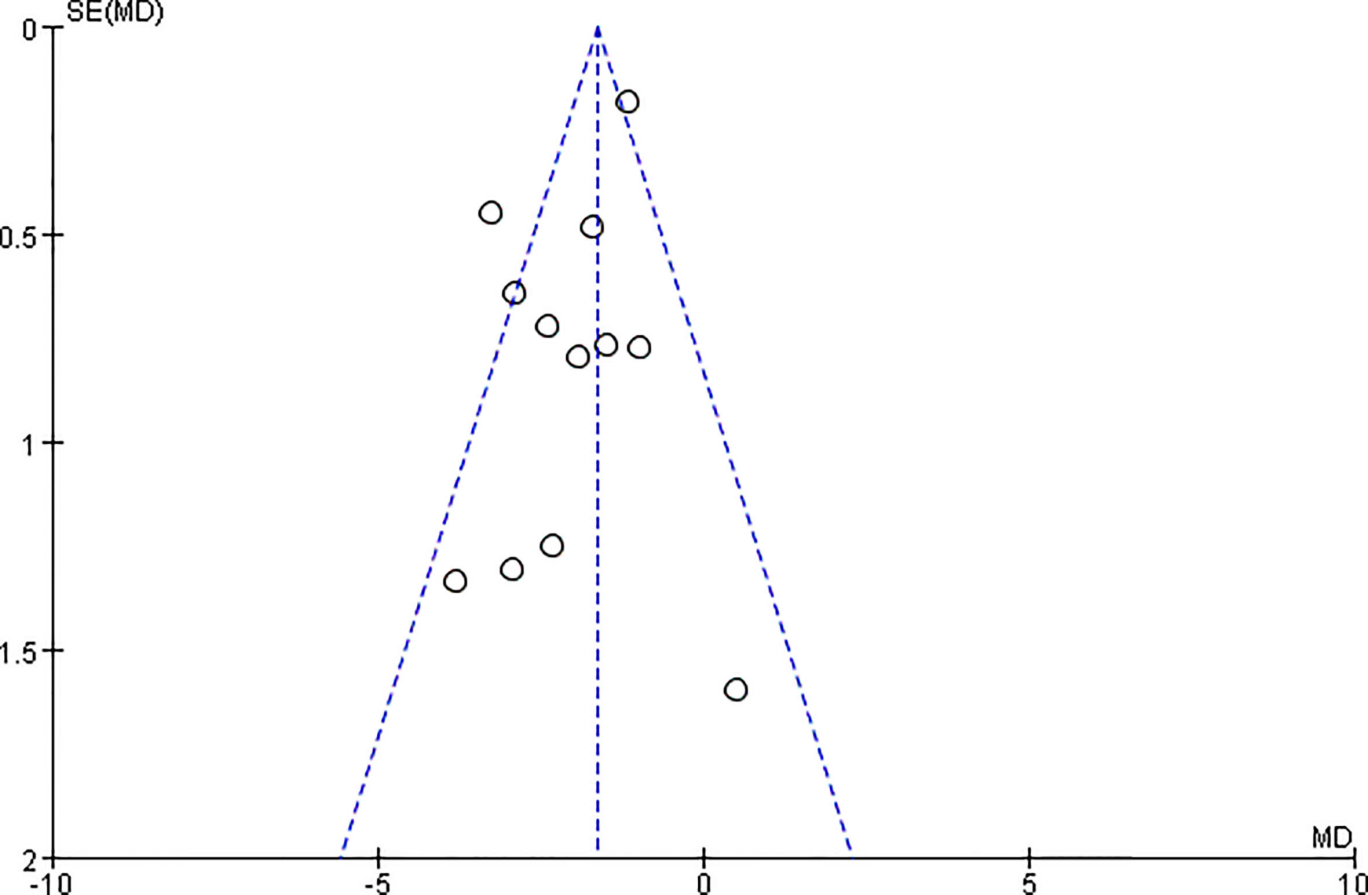
Funnel plots of the body mass index (BMI).

The publication bias of outcomes was evaluated using funnel plots based on the 10 studies. It can be seen that the funnel plot was not symmetrical, which indicated that no significant publication bias existed (**Figure 10**).

### 3.6 Grading of Recommendations Assessment, Development and Evaluation (GRADE) for the main outcomes

GRADE assessment was conducted for the main outcomes, and the results showed that the evidence quality of BW, BMI, HC, BFM and WHR was low; the evidence quality of WC was moderate (**Table 3**).

**Table 3:** Summary of GRADE.

Quality assessment							Number of patients		Effect	Quality	Importance
Outcomes	Study design	Risk of bias	Inconsistency	Indirectness	Imprecision	Publication bias	TCE group	Control group	Illustrative comparative risks (95% CI)		
BW	RCT	Serious^a^	Serious^b^	No serious	No serious^c^	Undetected	241	246	6.1 lower (8.79 to 3.42 lower)	⊕⊕⊝⊝ LOW^a,b,c^	IMPORTANT
BMI	RCT	Serious^a^	Serious^b^	No serious	No serious	Undetected	348	353	0 higher (1.91 to 1.36 lower)	⊕⊕⊝⊝ LOW^a,b^	IMPORTANT
BFM	RCT	Serious^a^	No serious	No serious	Serious^c^	Undetected	111	116	3.12 lower (4.49 to 1.75 lower)	⊕⊕⊝⊝ LOW^a,c^	IMPORTANT
WC	RCT	Serious^a^	No serious	No serious	No serious	Undetected	215	215	3.46 lower (4.67 to 2.24 lower)	⊕⊕⊕⊝ MODERATE^a^	IMPORTANT
HC	RCT	Serious^a^	No serious	No serious	Serious^c^	Undetected	85	85	2.94 lower (4.57 to 1.3 lower)	⊕⊕⊝⊝ LOW^a,c^	IMPORTANT
WHR	RCT	Serious^a^	No serious	No serious	Serious^c^	Undetected	120	120	0.04 lower (0.06 to 0.03 lower)	⊕⊕⊝⊝ LOW^a,c^	IMPORTANT

a Lacking blinding, randomization, or unclear allocation.

b Substantial heterogeneity.

c Small sample size.

## 4 Discussion

Obesity has been a more outstanding issue during the coronavirus disease 2019 (COVID-19) lockdown, which should be paid more attention to and adopt measures to control. It is the cause of many cardiovascular and cerebrovascular diseases (34) and may be accompanied by an increase in the prevalence of diabetes, cancer, and other diseases (35). There is no doubt that patients with simple obesity can lose weight through exercise, thereby reducing diseases caused by obesity. However, studies reported that the excessive exercise load was not suitable for obese people. They cannot bear too much intensity training (11, 36). TCE is a therapeutic physical and mental aerobic exercise that has been used to improve physical and mental health in China for thousands of years (20, 37–39), which does not need too much space and could be taken even in the COVID-19 lockdown. This meta-analysis and systematic review provide a quantitative estimate of whether TCE is a significant effective strategy for obesity improvement.

We have shown that TCE works significantly in decreasing BW, BMI, BFM, WC, HC, and WHR, which means it plays a positive role in weight reduction and weight loss maintenance. These effects are consistent with the results of individually included RCTs. For different types of TCE, subgroup analyses indicated that there was no significant difference among tai chi, Baduanjin, Yijinjing, and Wuqinxi in BW and BMI. And there was no significant difference among duration time, whether the time is more than 16 weeks or less; they both work. Many studies reported other outcomes, such as triglycerides, serum total cholesterol, low-density lipoprotein cholesterol, high-density lipoprotein cholesterol, and blood pressure (34, 40). Obesity is often accompanied by lipoprotein atherogenicity, diabetes, hypertension, and hyperlipidemia (41), thus when controlling and treating obesity, TCE can reduce these indicators at the same time. (25, 26). Safety was not reported as an outcome because this outcome was not discussed in these studies. It should be recorded as an outcome in future research.

Our study has several strengths. Firstly, a detailed search strategy is employed by searching different kinds of databases and trial registries. Moreover, no language limitation is set to ensure inclusion of as much data as possible from appropriate studies. Additionally, we try to find heterogeneity between the studies included in our study and to decrease it, which make the results more convincing. Furthermore, our study is the first systematic review and meta-analysis to evaluate the efficacy of TCE on obesity; it could provide evidence for clinical workers that would help them make better clinical decisions. Moreover, we offer ways to control weight at home for those with obesity, which is very necessary in the background of the lockdown.

Our review has several limitations as well. First, the sample size of this meta-analysis was relatively small. As a result, the unknown risk of bias caused by incomplete data could constrain our results. Second, the research results are not restricted by the language of the published articles. Moreover, the intervention of the intervention group of the study is TCE, which is from China, thus eight of the studies are Chinese, which may be of relatively low quality. Third, the intervention protocol varied significantly in the aspect of exercise type (tai chi, Baduanjin, qigong, Yijinjing, Wuqinxi), duration time (from 10 weeks to 12 months), frequency (1–14 times per week), continued time of each exercise (from 30 to 60 min), and at the same time, the control group received different interventions as well; this may be the reason for the heterogeneity of the article.

Despite these limitations, this meta-analysis provides information on the association between TCE and obesity.

## 5 Conclusion

In summary, the results of our meta-analysis indicated that TCE may have statistically significant effects on decreasing participants’ BW, BFM, BMI, HC, WC, and WHR, which indicated that it helps to control obesity. The findings presented in the meta-analysis may have important implications worldwide, especially in the COVID-19 lockdown. However, a precise and comprehensive conclusion calls for RCTs on a larger scale with more rigorous designs considering the inferior methodological quality and limited retrieved articles.

## Data availability statement

The original contributions presented in the study are included in the article/supplementary material. Further inquiries can be directed to the corresponding author.

## Author contributions

Data curation: ZY, YY, QX, and KH; Formal analysis: ZY and QG; Investigation: ZY and XW; Methodology: ZY, YY, and QX; Project administration: ZY; Resources: YY, ZY, and XW; Software: ZY and XW; Supervision: ZY, KH, and QG; Validation: YY and KH; Visualization: ZY and YY; Writing – original draft: ZY, KH, and YY; Writing – review and editing: ZY and XW. All authors contributed to the article and approved the submitted version.
